# Active Bleeding after Cardiac Surgery: A Prospective Observational Multicenter Study

**DOI:** 10.1371/journal.pone.0162396

**Published:** 2016-09-02

**Authors:** Pascal H. Colson, Philippe Gaudard, Jean-Luc Fellahi, Héléna Bertet, Marie Faucanie, Julien Amour, Yvonnick Blanloeil, Hervé Lanquetot, Alexandre Ouattara, Marie Christine Picot, ARCOTHOVA group

**Affiliations:** 1 Department of Anesthesiology and Critical Care Medicine, Arnaud de Villeneuve Academic Hospital, Montpellier University, Montpellier, France; 2 Institut de Génomique Fonctionnelle, Endocrinology Department, CNRS UMR 5203, INSERM U1191, University of Montpellier, 34094, Montpellier, France; 3 PhyMedExp, University of Montpellier, INSERM U1046, CNRS UMR 9214, 34295, Montpellier, cedex 5, France; 4 Department of Anesthesiology and Critical Care Medicine, Louis Pradel Academic Hospital, Lyon Bron, France; 5 Clinical Research and Epidemiology Unit, Academic Hospital, Montpellier, France; 6 Clinical Investigation Center, Academic Hospital, Montpellier, France; 7 Department of Anaesthesiology and Critical Care Medicine, Sorbonne University UPMC Univ Paris 06, UMR INSERM 1166 and Post-Genomic Platform, IHU ICAN, Paris, France; 8 Department of Anaesthesiology and Critical Care Medicine, Laënnec Academic Hôpital, Nantes, France; 9 Department of Anaesthesiology and Critical Care Medicine, Academic Hospital, Poitiers, France; 10 Department of Anaesthesiology and Critical Care Medicine II, Academic Hospital, Bordeaux-Pessac, France; Institut d'Investigacions Biomediques de Barcelona, SPAIN

## Abstract

**Main Objectives:**

To estimate the incidence of active bleeding after cardiac surgery (AB) based on a definition directly related on blood flow from chest drainage; to describe the AB characteristics and its management; to identify factors of postoperative complications.

**Methods:**

AB was defined as a blood loss > 1.5 ml/kg/h for 6 consecutive hours within the first 24 hours or in case of reoperation for hemostasis during the first 12 postoperative hours. The definition was applied in a prospective longitudinal observational study involving 29 French centers; all adult patients undergoing cardiac surgery with cardiopulmonary bypass were included over a 3-month period. Perioperative data (including blood product administration) were collected. To study possible variation in clinical practice among centers, patients were classified into two groups according to the AB incidence of the center compared to the overall incidence: “Low incidence” if incidence is lower and “High incidence” if incidence is equal or greater than overall incidence. Logistic regression analysis was used to identify risk factors of postoperative complications.

**Results:**

Among 4,904 patients, 129 experienced AB (2.6%), among them 52 reoperation. Postoperative bleeding loss was 1,000 [820;1,375] ml and 1,680 [1,280;2,300] ml at 6 and 24 hours respectively. Incidence of AB varied between centers (0 to 16%) but was independent of in-centre cardiac surgical experience. Comparisons between groups according to AB incidence showed differences in postoperative management. Body surface area, preoperative creatinine, emergency surgery, postoperative acidosis and red blood cell transfusion were risk factors of postoperative complication.

**Conclusions:**

A blood loss > 1.5 ml/kg/h for 6 consecutive hours within the first 24 hours or early reoperation for hemostasis seems a relevant definition of AB. This definition, independent of transfusion, adjusted to body weight, may assess real time bleeding occurring early after surgery.

## Introduction

Bleeding, as a source of anemia or blood transfusion, is a major complication after cardiac surgery [1–5]. Scientific societies have dealt repeatedly with blood conservation strategy in cardiac surgery and guidelines to improve perioperative blood management have been published [6,7]. However some relevant issues remained unsolved [8].

Old studies considered pivotal the bleeding flow from the chest drainage, mainly to decide to re-operate in emergency [9,10], otherwise, bleeding is usually quantified by the volume of packed red blood cells (PRBC) transfused [11,12]. The quantification depends therefore on the transfusion strategy, including the threshold for PRBC transfusion, which may vary largely from one center to another [12].

More attention has been paid to the chest tube flow in recent studies [5,13,14]. The upper tenth decile of the distribution of volume collected from the chest tube drainage over the first 12 hours after adult cardiac surgery was used to defined massive bleeding population in a large retrospective study [5]; a concept that has been included in the Universal Definition of Perioperative Bleeding (UDPB) proposed by an international expert group in 2014, along with 8 other variables, to classify bleeding in 4 levels of increasing severity [14]. Still, the UDPB classification is based mainly on the number of PRBC transfused, which counts for more than 50% in the ranking of mild or moderate classes [14]. Moreover, if massive bleeding and massive transfusion are quite appropriately defined, there is more uncertainty about moderate bleeding [14].

Given the limited published evidence on the postoperative chest tube output as a criterion of active bleeding, a group of French cardiac anesthetists and surgeons has decided to study the bleeding flow in chest tube over a short period of time in a population of cardiac surgery patients, through a prospective observational multicenter national survey. The main objective was to estimate the incidence of postoperative active bleeding (AB) based on a bleeding flow threshold through chest tubes drainage. Secondary objectives were first to analyze the AB characteristics and its management, to study the possible impact of routine clinical practice on AB incidence, and to identify factors of postoperative complications.

## Patients and Methods

### Study design and population

In this prospective observational multicenter national study, all adult patients (> 18 years) consecutively scheduled for undergoing elective or emergent cardiac surgery with cardiopulmonary bypass (CPB) who have faced an AB in 29 French centers (including private and public activity) were included from 1st October to 31st December 2010. Owing on an annual national activity around 35,000 to 40,000 cardiac surgery cases/year and an AB incidence of 3% we anticipated up to 300 cases during the survey.

The study protocol was approved by the national IRB Committee (Ministry of Higher Education and Research, Direction Générale pour la Recherche et de l'Innovation, Comité Consultatif sur le Traitement de l'Information en Matière de Recherche dans le domaine de la Santé, DRGI CCTIRS N° 10.230). Patients were formally informed by a descriptive written notice, but the need of written informed consent was waived by regulatory authorities because of the observational nature of the study.

### Definition of active bleeding

A steering group of cardiac anesthesiologists and surgeons of French centers designed a methodology to define AB independently of transfusion volume. Available published evidence related to bleeding after cardiac operations were reviewed [1, 6, 9–14]; bleeding exceeding 1.5 ml/kg/h for 6 consecutive hours within the first 24 hours has been selected. In addition, patients who were reoperated for bleeding within the first 12 hours were also considered as AB patients.

### Data collection

All data were collected on a standardized case-report form centralized in the Clinical Research and Epidemiology Unit of the Academic Hospital of Montpellier, France.

#### Preoperative data

Demographics data, medical history, organ function as it is usually assessed, and active treatment for coagulation were collected. Preoperative blood sample collected in routine practice allowed to get the blood count parameters and hemostasis function.

#### Peroperative data

The type of referral, the indication for the surgery and its context were collected. Surgery and cardiopulmonary bypass (CPB) were carried out at the discretion of medical teams, in agreement with national guidelines. Administration of blood products such as PRBC, fresh frozen plasma (FFP), platelets and fibrinogen concentrate as well as anticoagulation protocol were also recorded.

#### Postoperative data

Biological data including hemoglobin, platelets, fibrinogen, coagulation tests were collected upon arrival in the intensive care unit (ICU). The blood losses during the 6 hours of AB (H6) and after 24 hours in the ICU (H24) were recorded. AB was managed according to customary practices. Administration of blood products, hemostatic treatments and requirement of revision surgery were recorded. Hemodynamic instability during the AB was defined by the need of hemodynamic support (introduction of inotrope or vasopressor or increase in administered dose).

Monitoring of the patients in the ICU allowed us to evaluate the consequences of AB or its treatment on the immediate postoperative course, including death or complications. Postoperative complications were defined as: cardiac dysfunction if 2 inotropes or cardiac assistance were required, renal failure requiring dialysis, pulmonary dysfunction if ventilation was maintained more than 24 hours or if PaO2/FiO2 ratio was <200, liver dysfunction if plasma hepatic enzymes or bilirubin exceeded 2 fold the standard value, and hematologic dysfunction if platelet count was less than 80 G/L. Simplified Acute Physiology Score (SAPS) II within the first 24 h in ICU was collected to assess disease severity on ICU admission.

### Statistical analysis

Continuous variables were described in the study population with mean and standard deviation (SD) or median and first and third quartiles [Q25-Q75]. Categorical variables were described with frequencies and percentages.

Incidence was calculated by dividing center’s AB number declared by center’s cardiac surgical cases number during inclusion period. To study possible impact of a variation in the management of AB, patients were classified into two groups according to the AB incidence of their center: “Low incidence” if incidence of center is lower than overall incidence and “High incidence” if incidence of center is equal or greater than overall incidence.

Student or Mann-Whitney rank sum tests for quantitative variables and Chi-square or Fisher’s exact tests for categorical variables were used to determine differences in unadjusted preoperative, peroperative and postoperative characteristics between “Low incidence” and “High incidence” centers.

For taking into account center’s effect, associations between these characteristics and group (“Low incidence” vs “High incidence”) were tested using multivariate linear or logistic (according to type of dependent variable) mixed models. Patients’ characteristics were modeled with group (“Low incidence” vs “High incidence”) as fixed effect and center as random effect. Adjusted odds ratios (OR) or mean difference and their confidence interval 95% were presented [15].

Logistic regression was used to identify predictive factors of the occurrence of one or more postoperative complications. Univariate and multivariate analyses were carried out in each of the following groups of variables: preoperative characteristics, surgery characteristics, postoperative factors (at the arrival in ICU), context of the AB and postoperative management. Only variables clinically relevant were included in the model (for example details of preoperative antiplatelet treatment were not considered and postoperative blood products were only considered as continuous variables (number of units), and not as categorical variables. A backward procedure with entry level of 0.2 and removal level of 0.2 has been applied. In a second step, the variables selected in each analysis were entered in a final multivariate model and clinically relevant interactions (in particular emergency surgery and postoperative PRBC, emergency and acidosis) were proposed into the model. To determine the final model a backward selection with a removal level of 0.10 was used. Missing data have not been compensated by imputation method provided because they were not numerous. For the quantitative variables, the hypothesis of log linearity was verified. The absence of colinearity between variables was verified and in case of quasi-separation, the Firth penalized likelihood approach was used [16]. Odds-ratio (OR) and their 95% confidence intervals (CI) were calculated. The goodness-of-fit of the models was assessed using the Hosmer and Lemeshow chi-square test.

Statistical analyses were performed at the conventional two-tailed α level of 0.05 using SAS version 9.1 (SAS Institute, Cary, NC).

## Results

### Study population

During the survey period, 4904 patients underwent cardiac surgery in the 29 centers, and 129 experienced an AB (overall incidence 2.6%, 95%CI: [2.1; 3.1]) (Table 1). Incidence of AB varied from 0 to 16% between centers (Table 1). No significant correlation was observed between the number of cardiac surgery cases performed and the number of AB reported during the study period in each center.

**Table 1:** Incidence of active bleeding by center (*N = 29)*. Incidence of active bleeding (AB) during the 3-month period of the survey, according to the number (N) of cardiac surgery cases in each cardiac surgery center

Cardiac surgery center	Cardiac Cases (N)	AB Frequency (N)	AB Incidence (%)
**1**	337	18	5.34
**2**	268	1	0.37
**3**	254	19	7.48
**4**	118	3	2.54
**5**	70	2	2.86
**6**	74	5	6.76
**7**	98	3	3.06
**8**	108	1	0.93
**9**	332	4	1.20
**10**	141	4	2.84
**11**	92	15	16.30
**12**	151	3	1.99
**13**	168	5	2.98
**14**	125	3	2.40
**15**	150	5	3.33
**16**	124	2	1.61
**17**	238	4	1.68
**18**	214	2	0.93
**19**	159	2	1.26
**20**	106	4	3.77
**21**	96	1	1.04
**22**	90	1	1.11
**23**	226	1	0.44
**24**	168	1	0.60
**25**	94	0	0.00
**26**	259	4	1.54
**27**	198	5	2.53
**28**	243	5	2.06
**29**	203	6	2.96
**Overall**	4904	129	2.63

According to the overall incidence (2.6%), 43 (33%) patients were classified in “Low incidence” and 86 (67%) in “High incidence”.

Preoperative characteristics of patients with AB are presented in Table 2.

**Table 2:** Preoperative characteristics (*N = 129)*. Values are presented as N(%) or Median [Q25;Q75]. Significant difference between “Low incidence” and “High incidence” are denoted by *p<0.10, **p<0.05 and ***p<0.01, tested by Student /Mann-Whitney tests or Chi^2^/Fisher tests.

	All	“Low incidence” *N = 43*	“High incidence” *N = 86*	Adjusted OR or mean difference ^1^ [IC95%]
**Preoperative characteristics**				
Age *(years)*	70 [62;77]	65 [58;76]	72 [63;78] *	-2.46 [-8.01;3.10]
Weight *(kg)*	72 [64;79]	72 [68;85]	71 [63;76] ***	7.18 [0.95;13.42] ^**$$**^
BSA *(m*^*2*^*)*	1.79 [1.68;1.91]	1.85 [1.74;2.01]	1.75 [1.64;1.88] ***	0.13 [0.04;0.22] ^**$$$**^
Sex: Males	95 (74%)	34 (81%)	61 (71%)	0.57 [0.21;1.52]
Diabetes	22 (17%)	10 (23%)	12 (14%)	2.04 [0.71;5.89]
Hypertension	82 (64%)	31 (72%)	51 (59%)	1.77 [0.79;3.96]
LVEF < 40%	14 (11%)	5 (12%)	9 (10%)	1.13 [0.35;3.64]
**Preoperative treatment**				
Anticoagulant	39 (30%)	14 (33%)	25 (29%)	1.23 [0.51;2.93]
Antiplatelet	87 (67%)	29 (67%)	58 (67%)	1.00 [0.45;2.21]
**Hemobiology & Coagulation tests**				
Hemoglobin *(g/dL) (SV*:*12–16 g/dL)*	13.5 [12.4;14.7]	13.5 [12.4;14.9]	13.5 [12.3;14.7]	0.06 [-0.57;0.69]
Platelet *(g/L) (SV*: *150–400 g/L)*	207 [168;242]	207 [157;240]	208 [169;243]	0.23 [-29.47;29.92]
Fibrinogen *(g/L) (SV*: *1*.*9–4 g/L)*	3.5 [3.0;4.2]	3.6 [3.0;4.6]	3.5 [3.0;4.0]	0.42 [-0.06;0.89] ^**$**^
aPTT ratio *(SV < 1*.*2)*	1.00 [0.9;1.10]	1.03 [0.96;1.15]	1.00 [0.93;1.06] *	-0.01 [-0.19;0.17]
PT *(%) (SV*:*80–100%)*	87 [79;97]	93 [79;100]	86 [77;95]	1.99 [-4.36;8.35]

^1^ Significant mean difference or OR between “Low incidence” and “High incidence” adjusted for center are denoted by $ p<0.10, $ $ p<0.05 and $ $ $ p<0.01. OR: Odds ratio; BSA: body surface area; LVEF: Left ventricular ejection fraction; VKA: vitamin K antagonist; UFH: unfractionated heparin; LMWH: low-molecular-weight heparin; aPTT: activated partial thromboplastin time; PT: Prothrombin Time; SV: standard value.

The surgery consisted of a combination of at least 2 surgical procedures in 32 patients (25%) out of the 129 patients. Heparin was used as anticoagulant and antagonized by protamine in all cases. One hundred and twelve patients (88%) received tranexamic acid (3.2 ±3.4 g) and this administration was significantly different between”Low incidence” and “High incidence” (p = 0.02) (Table 3). Sixty five patients (50%) received blood products in the operating room, in similar proportion in the 2 groups, “Low incidence” and “High incidence”, except for FFP (p = 0.05). However, these differences are mitigated when the center effect is taken into account (Table 3)

**Table 3:** Peroperative characteristics and biology at the arrival in ICU (*N = 129)*. Values are presented as N(%) or Median [Q25;Q75]. Significant difference between “Low incidence” and “High incidence” are denoted by *p<0.10, **p<0.05 and ***p<0.01, tested by Student /Mann-Whitney tests or Chi^2^/Fisher tests.

	All	“Low incidence” *N = 43*	“High incidence” *N = 86*	Adjusted rate or mean difference ^2^ [IC95%]
**Surgery**				
Emergency	23 (18%)	7 (16%)	16 (19%)	1.27 [0.29;5.57]
Redo	15 (12%)	9 (21%)	6 (7%) **	3.61 [1.13;11.54] ^**$$**^
Endocarditis	8 (6%)	4 (9%)	4 (5%)	2.85 [0.44;18.42]
Type				
∘ CABG	63 (49%)	21 (49%)	42 (49%)13 (15%)	1.07 [0.42;2.70]
∘ Mitral valve	18 (14%)	5 (12%)	40 (47%)	0.74 [0.24;2.26]
∘ Aortic valve	53 (41%)	13 (30%)	13 (15%)	0.50 [0.20;1.26]
∘ Others 1	21 (16%)	8 (19%)		1.26 [0.41;3.89]
CPB				
∘ Time *(min)*	92 [71;127	110 [75;150]	87 [68;126]	15.18 [-19.78;50.14]
∘ Hypothermia(<32°C)	6 (5%)	4 (9%)	2 (2%)*	4.15 [0.63;27.45]
∘ Circulatory arrest	3 (2%)	1 (2.3%)	2 (2%)	1.00 [0.09;11.69]
∘ ECMO post CPB	6 (5%)	6 (14%)	0 (0%) ***	-^3^
Transfusion				
∘ Over all	65 (50%)	22 (51%)	43 (50%)	1.01 [0.35;2.93]
∘ PRBC	58 (45%)	18 (42%)	40 (47%)	0.84 [0.29;2.39]
∘ FFP	19 (15%)	10 (24%)	9 (11%) **	3.12 [0.89;11.22] ^**$**^
∘ Platelets	29 (23%)	13 (31%)	16 (19%)	2.13 [0.56;8.10]
Hemostatic treatment				
∘ Fibrinogen	6 (5%)	2 (5%)	4 (5%)	1.07 [0.18;6.29]
∘ Tranexamic acid	112 (88%)	41 (98%)	71 (83%) **	2.82 [0.12;65.12]
**Biology at the arrival in ICU**				
Hemoglobin *(g/dL) (SV = 12–16 g/dL)*	10.6 [9.6;11.8]	10.4 [9.2;11.8]	10.70 [9.6;12.0]	-0.13 [-1.02;0.76]
Platelet *(g/L) (SV = 150–400 g/L)*	120 [98;151]	135 [111;158]	114 [92;146] **	14.75 [-9.17;38.68]
Fibrinogen *(g/L) (SV = 1*.*9–4 g/L)*	1.9 [1.6;2.3]	2.0 [1.6;2.8]	1.8 [1.5;2.2] *	0.43 [-0.09;0.95]
aPTT ratio *(SV < 1*.*2)*	1.19 [1.04;1.44]	1.24 [1.00;1.50]	1.18 [1.09;1.44]	0.17 [-0.41;0.74]
PT *(%) (SV = 80–100%)*	56 [46;63]	59 [48;64]	55 [46;62]	3.94 [-1.95;9.83]

^1^ heart assistance, heart transplant, thoracic aorta surgery, others.

^2^ Significant mean difference or OR between “Low incidence” and “High incidence” adjusted for center are denoted by $ p<0.10, $ $ p<0.05 and $ $ $ p<0.01

^3^ Impossible to estimate as number = 0. OR: Odds ratio; CABG: coronary artery bypass graft; CPB: Cardiopulmonary bypass; ECMO: extracorporeal membrane oxygenation; PRBC: packed red blood cell unit; FFP: fresh-frozen plasma unit; SV: standard value; aPTT: activated partial thromboplastin time; PT: Prothrombin Time

### Active bleeding characteristics

Among the 129 patients, 77 (60%) were included because of excessive bleeding (≥1.5 ml/kg/h for 6 consecutive hours), and 52 (40%) for early reoperation. The median delay between admission to ICU and the start of AB was 5.5 [3.0;6.8] hours. Forty-four patients (34%) had hemodynamic instability during AB.

Early reoperation (N = 52) was decided upon the bleeding volume (N = 48, 92%), or tamponade (N = 4, 8%) with a median delay between admission to ICU and reoperation of 4.8 [2.3; 6.0] hours. The rate of early reoperation was 48% (N = 21) in “Low incidence” vs 36% (N = 31) in “High incidence” (p = 0.16) mainly upon the bleeding volume in both groups (90% vs 94%, respectively, p = 1.00). The median delay between admission to ICU and reoperation for hemostasis was significantly different between the two groups (respectively, 5.6 [4.0; 7.9] hours vs 4.0 [1.5; 5.1] hours, p = 0.01) when the center effect is not taken into account.

Patients who were included because of excessive bleeding (N = 77), had median H6 blood loss of 1,000 [820;1,375] ml. It was 1,020 [1,028;1,665] ml and 970 [800;1,285] ml in “Low incidence” (N = 22) and “High incidence” (N = 55), respectively (p = 0.01).

Median H24 blood loss for all patients (N = 129) was 1,680 [1,280; 2,300] ml, without significant differences between “Low incidence” and “High incidence” (1,685 [1,200; 2,610] ml vs 1,670 [1,340; 2,208] ml, respectively, p = 0.97).

### Management and follow-up in ICU

Most patients (95%) received at least one blood product, 88% received PRBC units, but only 24% received 5 PRBC units or more; the median number of PRBC units transfused for the first 24 hours including the peroperative period was 3 [2;5] (Table 4). Point-of-care devices, Thromboelastograph (TEG) (Haemoscope; Niles, IL, USA) or ROTEM (Pentapharm, Munich, Germany), were used for thromboelastometric analyses in 18 patients (14%), without significant difference between “Low incidence” and “High incidence” (p = 0.11). There were no significant differences between these two groups, except for tanexamic acid given a in higher proportion of patients in “Low incidence” (Table 4).

**Table 4:** Postoperative management during the first 24 hours. Values are presented as N(%) or Median [Q25;Q75]. Significant difference between “Low incidence” and “High incidence” are denoted by *p<0.10, **p<0.05 and ***p<0.01, tested by Student /Mann-Whitney tests or Chi^2^/Fisher tests.

	All	“Low incidence” *N = 43*	“High incidence” *N = 86*	Adjusted rate or mean difference ^1^ [IC95%]
**Point-of-care coagulation tests**	18 (14%)	3 (7%)	15 (17%)	1.13 [0.04;31.61]
**Hemostatic treatments**				
Protamine	56 (43%)	23 (53%)	33 (38%)	1.95 [0.68;5.55] -
∘ Dose (mg)	50 [50;100]	50 [50;100]	50 [50;100]	-20.48 [55.29;14.33]
Tranexamic acid	15 (12%)	13 (30%)	2 (2%) **	14.11 [1.45;137.34] ^**$$**^
∘ Dose (g)	2 [1;3]	2 [1;2]	2 [1;3]	0.27 [-1.97;2.51]
Desmopressin	8 (6%)	5 (12%)	3 (3%)	2.77 [0.25;30.91]
∘ Dose (μg)	20 [16;25]	16 [15;20]	21 [20;28]	-6.56 [-41.02;27.91]
Prothrombin complex	11 (9%)	6 (14%)	5 (6%)	2.51 [0.37;17.17]
Dose (ml)	40 [20;40]	40 [20;40]	40 [30;50]	-6.06 [-40.34;28.23]
Factor VIIa	4 (3%)	3 (7%)	1 (1%)	6.25 [0.57;68.16]
∘ Dose (mg)	6 [5;7]	5 [5;7]	7 [7;7]	-1.33 [-7.07;4.40]
**Blood products**				
Over all	122 (95%)	42 (98%)	80 (94%)	2.38 [0.23;24.67]
PRBC	113 (88%)	38 (88%)	75 (87%)	1.01 [0.32;3.22]
∘ Dose *(unit*, *284 ±28 ml/ unit)*	3 [2;5]	4 [2;6]	2 [2;4] **	1.59 [0.60;2.58] ^**$$$**^
FFP	98 (77%)	38 (88%)	60 (71%) **	3.17 [1.10;9.10] ^**$$**^
∘ Dose *(unit*, *200–300 ml/unit)*	4 [2;6]	3 [2;6]	4 [3;6]	0.17 [-1.59;1.93]
Platelets	61 (48%)	27 (64%)	34 (40%) ***	2.42 [0.71;8.32]
∘ Dose *(1011)*	5 [2;7]	5 [3;8]	6 [1;7]	0.15 [-2.82;3.12]
Fibrinogen	39 (31%)	11 (26%)	28 (33%)	0.91 [0.30;2.73]
∘ Dose *(g)*	3 [2;3]	3 [2;3]	3 [2;3]	1.06 [-1.15;3.27]

^1^ Significant mean difference or OR between “Low incidence” and “High incidence” adjusted for center are denoted by $ p<0.10, $ $ p<0.05 and $ $ $ p<0.01. OR: Odds ratio; PRBC: packed red blood cell unit; FFP: fresh-frozen plasma unit

Out of the 77 patients, a delayed reoperation was decided for 28 patients (36%). The resternotomy was performed thus with a median delay of 12.4 [8.8;18.7] hours from arrival in ICU. The delayed reintervention occurred in 41% (N = 9) vs 35% (N = 19) for “Low incidence” and “High incidence”, respectively (p = 0.60).

Median ICU stay was 4 [3; 7] days without significant difference between “Low incidence” and “High incidence” (5 [3; 7] days vs 4 [3; 6] days, respectively, p = 0.32). Fifty-eight patients (45%) had at least one postoperative complication. Prevalence was 68% for pulmonary dysfunction, 50% for thrombopenia, 37% for cardiac dysfunction, 22% for renal failure and 15% for liver dysfunction; mean SAPS II score was 34.9 ±13.5.

The incidence of death in the ICU was 7%. All patients who died in ICU had significantly more complications than patients who survived, specifically cardiac failure (89% vs 27%, p<0.001), acute renal failure (56% vs 14%, p = 0.01), liver dysfunction (44% vs 10%, p = 0.02) and septic complications (67% vs 26%, p = 0.02).

Patients undergoing resternotomy (N = 80) had more pulmonary dysfunction (79% vs 50%, p = 0.02) and tended to have a longer intubation time (21 [12;48] vs 16 [9;24] hours, p = 0.07) but had similar length of stay in ICU (5 [3;7] vs 4 [3;7] days, p = 0.79) compared with patients who did not have resternotomy (N = 49).

The multivariate analysis of risk factors of occurrence of at least one complication showed that BSA greater or equal than 1.68, preoperative creatinine, emergency surgery, postoperative PT and acidosis and number of PRBC transfused in ICU were significant risk factors of postoperative complication. Interactions were not significant (Table 5).

**Table 5:** Risks factors of occurrence of at least one postoperative complication. Univariate and multivariate analyses.

	Univariate analysis			Multivariate analysis by blocks of variables			Multivariate analysis *(N = 111)*		
	OR	[95% CI]	p	OR	[95% CI]	p	OR	[95% CI]	p
**Preoperative factors (block 1)**				***N = 120***					
BSA *(m*^*2*^*)*			0.01			0.04	**-**		**0.08**
∘ < 1.68	1			1			**1**		
∘ [1.68;1.91]	3.12	[1.18;8.27]	0.02	3.29	[1.07;10.12]	0.04	**3.37**	**[0.90;12.61**	**0.07**
∘ > = 1.91	5.28	[1.77;15.75	<0.01	4.91	[1.37;17.59]	0.01	**5.60**	**[1.19;26.46]**	**0.03**
Sex (*Male vs Female)*	4.13	[1.63;10.43]	<0.01	-	-	-	-	-	-
LVEF < 40% *(Yes vs No)*	3.49	[1.03;11.78]	0.04	-	-	-	-	-	-
Creatinine *(μmol/L)*	1.03	[1.01;1.04]	<0.001	1.03	[1.01;1.04]	<0.01	**1.02**	**[1.00;1.04]**	**0.02**
Platelet (*g/L)*	1.00	[0.99;1.00]	0.14	-	-	-	-	-	-
PT *(%)*	0.97	[0.95;0.99]	0.01	0.98	[0.95;1.00]	0.07	-	-	-
**Peroperative factors** * **(block 2)**				***N = 129***					
Emergency *(Yes vs No)*	5.94	[2.05;17.25]	<0.01	4.97	[1.71;14.40]	<0.01	**4.20**	**[1.19;14.79]**	**0.03**
Hypothermia (< 32°C) *(Yes vs No)* *	17.70	[0.78;403.40]	0.07	-	-	-	-	-	-
ECMO *(Yes vs No)* *	17.70	[0.78;403.40]	0.07	13.97	[0.55;354.49]	0.11	-	-	-
**Postoperative factors (block 3)**				***N = 108***					
Delay between end of surgery and ICU admission *(hours)*	1.70	[1.16;2.51]	<0.01	1.70	[1.07;2.70]	0.02	-	-	-
PT *(%)*	0.96	[0.92;0.99]	<0.01	0.94	[0.91;0.98]	<0.01	**0.95**	**[0.91;1.00]**	**0.03**
aPTT ratio	1.88	[0.78;4.51]	0.16	-	-	-	-	-	-
**AB context (block 4)**				***N = 112***					
Delay between admission to ICU and the start of AB *(± 5 hours vs > 5 hours)*	1.62	[0.79;3.33]	0.19	-	-	-	-	-	-
Acidosis (pH ≤ 7.3) *(Yes vs No)*	7.56	[2.06;27.67]	<0.01	3.94	[0.97;16.02]	0.06	**6.80**	**[1.52;30.38]**	**0.01**
Temperature *(36°C vs [36°C;38*.*2°C])*	2.51	[0.87; 7.28]	0.09	2.31	[0.70;7.63]	0.17	-	-	-
Hemodynamic instability *(Yes vs No)*	6.05	[2.70;13.58]	<0.0001	4.28	[1.67;11.00]	<0.01	-	-	-
Resuming anticoagulation *(Yes vs No)*	3.04	[0.79;11.66]	0.10	-	-	-	-	-	-
**Postoperative management factors** * **(block 5)**				***N = 118***					
Bleeding volume at H24	1.00	[1.00;1.00]	0.02	-	-	-	-	-	-
Protamine *(No vs Yes)*	1.95	[0.96;3.99]	0.07	2.86	[1.25;6.54]	0.01	-	-	-
Desmopressin *(No vs Yes)*	6.23	[0.74;52.23]	0.09	-	-	-	-	-	-
Albumin *(Yes vs No)*	1.68	[0.81;3.49]	0.17	-	-	-	-	-	-
Factor VIIa (Yes vs No)*	11.80	[0.44;315.31]	0.14	-	-	-	-	-	-
Blood product administration *(Yes vs No)* *	11.40	[0.50;259.74]	0.13	-	-	-	-	-	-
PRBC (N)	1.38	[1.14;1.66]	<0.001	1.40	[1.16;1.71]	<0.001	**1.27**	**[1.00;1.61]**	**0.05**
FFP (N)	1.14	[1.01;1.28]	0.03	-	-	-	-	-	-
Platelets (10^11^)	1.13	[1.02;1.25]	0.02	-	-	-	-	-	-
Fibrinogen (g)	1.30	[1.00;1.69]	0.05	-	-	-	-	-	-

* Estimation with Firth’s Penalized Likelihood approach. Hosmer-Lemeshow p-value = 0.59. BSA: body surface area; LVEF: Left ventricular ejection fraction; aPTT: activated partial thromboplastin time; PT: Prothrombin Time; ECMO: extracorporeal membrane oxygenation; AB: active bleeding; PRBC: packed red blood cells; FFP: fresh-frozen plasma

## Discussion

To our knowledge, this multicenter study is the first attempt to identify active bleeding after cardiac surgery in a dynamic way. Using a definition of 1.5 ml/kg/hour blood loss during a 6-hour period, or early reoperation, the overall incidence is 2.6%. We observed inter-center variability among the 29 centers participating in the survey, but no relationship between the center activity and the AB incidence.

Previous studies on postoperative bleeding which used a surrogate for bleeding evaluation, PRBC transfusion, found that up to 10–15% patients received at least 4 PRBC units within the first 24 hours [2,11,12,17]. However, PRBC transfusion rate may not be an accurate estimate of bleeding, as transfusion strategy varies widely between centers [12,18–20]. Some recent studies have incorporated blood loss from chest drains to assess more directly bleeding, but they used a predefined and prolonged time of observation, not adjusted for body weight [5,14]. In the experts’ proposal of the UDPB, a blood loss over the first 12 postoperative hours was used to classify patients with 8 other criteria [14]. In their validation cohort, blood loss volume influenced the ranking only in 16.7% of all the bleeding patients, and even less, 4.4%, for the moderate class. Moreover, the patients were more frequently classified in moderate (24%) than mild (14%) bleeding categories; that questions the validity of the definition of these classes [14]. Taken together, these drawbacks suggest that UDPB fails to characterize mild/or moderate bleeding. One study used an hourly blood loss (2 ml/kg/hour) during a shorter period of time (3 hours or less) to define a bleeding score; 7.5% to 8.2% patients met the criterion [20]. The lower incidence of bleeding observed in our study may be explained by the longer time of observation (6 hours), which was deliberately decided in order to exclude patients with active bleeding easily resolved within few hours. However, as mentioned by the authors [20], blood loss is a better measure of early postoperative bleeding, reducing observer dependent error. Therefore, the hourly quantification of blood loss may improve the accuracy of postoperative bleeding measurement, furthermore in real time

The incidence of reoperation for surgical hemostasis (1.6%) is in the lower range of the rate usually reported (1 to 11%) [5,9,10]. Conversely, the proportion of the patients who underwent early reoperation among bleeding patients is quite high (40%). Of note, these patients were reoperated due to bleeding, meaning that the bleeding rate exceeded 1.5 ml/kg/hour, but the reoperation intervened before the 6-hour period elapsed. Revision surgery remains a major treatment for postoperative bleeding, but surgeons are reluctant to redo surgery because of the increased risk of postoperative complications [9,10]. Of course, intense bleeding with a short-term vital threat (hemorrhagic shock or tamponade) is not in question. More questionable is the timing in case of bleeding without serious hemodynamic instability. Previous studies have shown that adverse outcomes were more frequent when patients waited more than 12 hours for the resternotomy [9,10]. In the study, the median delay between arrival in ICU and the early revision surgery was less than 5 hours, showing that the decision to return to the operating room was not delayed. Reoperation has had a minimal impact on postoperative follow up without significant effect on the ICU stay. Indeed, in the multivariate analysis no element of bleeding management (surgery or hemostatic treatments) is significantly associated with the occurrence of postoperative complications.

Among factors of postoperative bleeding, besides surgical causes, fibrinogen plays a pivotal role [21–27]. In the present study, preoperative plasma fibrinogen concentration is lower than 3.8 g/l, which has been considered as a threshold for preventive administration of fibrinogen concentrate for some authors (25). Only 6 out of the 129 patients have received fibrinogen concentrate during surgery, when they required PRBC transfusion for active bleeding before surgery completion. We cannot exclude that prophylactic administration of fibrinogen would have prevented bleeding in these patients [24,25]. We observed a lower platelet count and a trend in lower plasma fibrinogen concentration at arrival in ICU in High incidence centers (Table 3). The functional consequences of low platelet count or low plasma fibrinogen concentration on coagulation would have been well monitored by point-of-care hemostasis devices [25–29]. The study was performed when these devices were used routinely in very few centers (3/29), before they became strongly recommended in guidelines [7,26]. Nevertheless, guidelines might have altered patient management during bleeding, but definitely not the relevance of the definition of active bleeding directly based on chest drain blood flow.

Several differences are observed between the 2 groups of AB incidence, though the differences should be considered with caution as the results are mitigated by the center effect analysis (Table 3). There is no clinically relevant difference regarding patient selection except for body weight or BSA, smaller in “High incidence”, which has been reported as a risk factor of bleeding or transfusion in several studies [18,21,30], Surgery characteristics were roughly the same between centers, but a higher proportion of redo surgery and ECMO, factors known to predict postoperative bleeding [18,21,30], were observed in “Low incidence”. Therefore, other factors should explain the higher AB incidence in “High incidence”. In this respect, lower use of tranexamic acid or FFP during surgery in the “High incidence” suggests differences in blood conservation strategy compared to “Low incidence”. Similarly, a lower proportion of patients received FFP, platelets and tranexamic acid, and a lower number of PRBC, and inversely, a higher proportion of patients had quicker early reoperation after surgery in “High incidence” compared to “Low incidence”; these characteristics suggest differences in strategy of bleeding treatment between the 2 groups [6,12].

### Study Limitations and Strengths

The study was designed to cover a short period of time to get an extensive and exhaustive collection of AB. Once the survey was completed, an independent control was done by crossing the data collected with the database from the Programme de Médicalisation des Systèmes d'Information (PMSI) used for price scale fixing. There is only a marginal difference (1.1%) between both sources for the number of cardiac cases and postoperative hemorrhage reports.

There is no gold standard measurement of active bleeding or validated AB definition so that it is difficult to compare our results to published data. Indeed the steering group reviewed literature available in 2009, and found no scientific evidence of active bleeding evaluation after surgery. Only a very old book reference mentioned a bleeding volume as threshold to decide reoperation [31]. The new definition (UDPB) was published in 2014 [14], but is not able to characterize bleeding in real time with good precision. Obviously, a realistic method for postoperative bleeding measurement was missing, which justifies the present study.

A possible confounding effect of the study design could be to induce to pay more attention to the bleeding cases. Nevertheless, it is doubtful that active bleeding would have been not taken into account appropriately without the survey.

The multicenter collection and the exhaustive data on the current management of active postoperative bleeding in a realistic cohort of patients are the main strengths of the study.

## Conclusion

Using chest tube blood loss with an hourly rate within the first postoperative hours seems quite relevant to identify active bleeding after cardiac surgery. Further studies are needed to assess whether the kinetic dimension of bleeding measurement may prompt bleeding treatment and eventually improve blood conservation strategy.
